# Nucleic Acids as Emerging Regulators of Calcium Phosphate Biomineralization

**DOI:** 10.1096/fj.202600820RR

**Published:** 2026-08-28

**Authors:** Fanny Duhalde, Jessem Landoulsi, Xavier Houard

**Affiliations:** ^1^ Sorbonne Université, INSERM, Centre de Recherche Saint‐Antoine, CRSA Paris France; ^2^ Sorbonne Université, CNRS, Laboratoire de Réactivité de Surface, LRS Paris France

**Keywords:** biomineralization, calcium phosphate, collagen, ectopic mineralization, extracellular vesicles, nucleic acids, pathology

## Abstract

Calcium phosphate biomineralization is a highly regulated process that underlies the formation of vertebrate mineralized tissues. While proteins have long been recognized as the principal regulators of mineral formation, accumulating experimental evidence suggests that nucleic acids may also contribute to this process through mechanisms that remain largely unexplored. Their negatively charged phosphate backbone, molecular structural versatility, and ability to interact with calcium phosphate phases make nucleic acids attractive candidates for regulating mineral nucleation, growth, and phase transformations. In this review, we first summarize the current understanding of physiological calcium phosphate biomineralization, with particular emphasis on the roles of collagen and matrix vesicles in mineral formation as well as factors influencing this process. We then examine the mechanism by which nucleic acids interact with calcium phosphate, highlighting experimental evidence demonstrating nucleic acid adsorption, coprecipitation, and mineral templating. Finally, we discuss the growing evidence supporting the presence of extracellular nucleic acids within mineralized tissues and ectopic mineralization, their diverse biological origins, and their potential contribution to pathological mineralization through mechanisms involving bacteria, extracellular vesicles, cell death, and neutrophil extracellular traps. These observations suggest that nucleic acids should be considered as potential contributors to calcium phosphate biomineralization. Although their precise physiological functions remain poorly understood, integrating nucleic acids into current mechanistic models may refine our understanding of both physiological and pathological mineralization processes, thus opening new avenues for biomarker discovery and therapeutic investigations.

## Graphical abstract

Calcium phosphate biomineralization has traditionally been considered a protein‐regulated process. This review highlights the emerging role of nucleic acids, which interact with mineral phases through adsorption, coprecipitation, and templating, thereby influencing crystal nucleation and growth. Under pathological conditions, these interactions may contribute to ectopic calcification in association with extracellular vesicles, apoptotic debris, and NETs. Incorporating nucleic acids into current models provides new opportunities for biomarker discovery and the development of targeted therapeutic strategies. Image generated using FigureLabs.

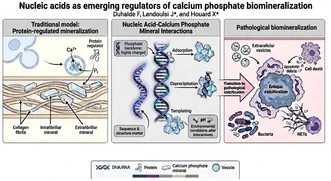

**Abbreviations**
ACP: amorphous calcium phosphate
Anx: annexin
ATP: adenosine 5′‐triphosphate
BSP: bone sialoprotein
CaOx: calcium oxalate
CaP: calcium phosphate
CaPP: calcium pyrophosphate
CfNAs: cell‐free nucleic acids
DMP1: dentin matrix protein 1
DSPP: dentin sialophosphoprotein
ECM: extracellular matrix
eDNA: extracellular DNA
ER: endoplasmic reticulum
EV: extracellular vesicles
FOP: fibrodysplasia ossificans progressive
GACI: generalized arterial calcification in infancy
HAP: hydroxyapatite
IDP: intrinsically disordered proteins
KCS1: Kenny‐Caffey syndrome 1
MEPE: matrix extracellular phosphoglycoprotein
MET: macrophage extracellular traps
MGP: matrix Gla protein
miRNA: micro RNA
mRNA: messenger RNA
MSU: monosodium urate
MVs: matrix vesicles
NCPs: non‐collagenous proteins
NET: neutrophil extracellular traps
NPP1: ecto‐nucleotide pyrophosphatase/phosphodiesterase 1
OA: osteoarthritis
OPN: osteopontin
PC: phosphocholine
Pi: inorganic phosphate
PPi: inorganic pyrophosphate
PXE: pseudoxanthoma elasticum
SIBLING: small integrin‐binding ligand, N‐linked glycoprotein
siRNA: small interfering RNA
SMC: smooth muscle cells
SMPD3: sphingomyelin phosphodiesterase 3
ssDNA: single‐stranded DNA
TEM: transmission electron microscopy
TNAP: tissue nonspecific alkaline phosphatase
XRD: X‐ray diffraction

## 1 Introduction

Biomineralization is a fundamental physiological process that evolved early in metazoans and has been the subject of extensive research, particularly in vertebrates. The emergence of mineralized tissues constitutes a major evolutionary innovation that has profoundly contributed to the diversification and evolutionary success of vertebrates. A pivotal event that marks vertebrate evolution is the development of hinged jaws and was accompanied by numerous morphological and phenotypic innovations [1]. This led to the dominance of Gnathostomes, which are divided into two groups that diverged approximately 450 million years ago [2]: cartilaginous fish and bony vertebrates. The origins and development of these evolutionary innovations, along with their role in forming different mineralized tissues, remain key unresolved questions. A notable hypothesis suggests that deuterostomes share an ancestral specialized “biomineralization toolkit” that provided the molecular basis of mineral formation [3]. Through evolutionary diversification, these ancestral molecular and structural components were progressively recruited and modified, leading to the emergence of increasingly complex skeletal tissues under highly precise biological regulation.

In humans, six types of mineralized tissues have been identified: bone, calcified cartilage, dental tissues including dentin, enamel, cementum, and otoconia. They differ in terms of composition, structure, morphology, and mechanical requirements, but they all contain a mineral phase consisting of calcium phosphate (CaP) in the first five and calcium carbonate in the otoconia [4, 5]. Physiological and physicochemical factors influencing the mineral formation have been the subject of a vast literature (see Refs [6, 7] and references therein), demonstrating the deployment of a variety of biomacromolecules capable of directing the mineralization process under tighter biological control in space and in time.

In light of the principles of the biomineralization toolkit, it becomes clear that the four major classes of biomolecules played a pivotal role in driving the emergence and evolution of hard mineralized tissues in vertebrates. Among them, proteins have been extensively investigated and are now recognized as the principal regulators of biomineralization. They stabilize amorphous mineral phases, control crystal nucleation and growth, inhibit pathological mineralization, promote mineral resorption, and coordinate multiple stages of the mineralization process [8]. Polysaccharides and lipids have also been implicated in physiological mineralization, although their roles remain considerably less explored than those of proteins [9, 10]. By contrast, nucleic acids have received little attention in the field of calcium phosphate biomineralization. Yet, several physicochemical and biological observations support their potential involvement in mineral formation. Their negatively charged phosphate backbone and structural diversity confer the ability to interact with calcium phosphate precursors, serve as templates for mineral nucleation and modulate phase transformations. Despite these physicochemical properties, the contribution of nucleic acids to calcium phosphate biomineralization remains poorly characterized, even though they could make nucleic acids key players in both physiological and pathological biomineralization.

This review examines the hypothesis that nucleic acids constitute previously overlooked regulators of calcium phosphate biomineralization. We first summarize the current understanding of physiological biomineralization and identify the molecular mechanisms governing physiological calcium phosphate nucleation and growth. We then review the physicochemical interactions between nucleic acids and calcium phosphate that provide a mechanistic basis for their involvement in biomineralization. Finally, we discuss emerging evidence supporting the presence and potential functions of extracellular nucleic acids in pathological mineralization, and highlight the key questions that must be addressed to establish their contribution to biomineralization.

## 2 Mechanisms of Biogenic Mineralization

Native mineralized tissues of vertebrates are composites made of soft biological matter and hard mineral phase entangled within various hierarchical multiscale structures [11, 12]. The mineral phase is mainly composed of poorly crystalline ion‐substituted hydroxyapatite (HAP), whose composition differs from stoichiometric HAP and influences its physicochemical reactivity [13]. This peculiar composition influences the mineral interactions with its surrounding environment, particularly its reactivity, such as dissolution or reprecipitation in aqueous solutions [14], as well as their interactions with proteins and cells [15]. Biogenic mineralization mainly occurs in the extracellular matrix (ECM), specifically within a dense network made of self‐assembled collagen and other biomacromolecules. Various types of collagens self‐assemble into supramolecular structures to meet different properties, depending on the physiological and mechanical requirements, thus contributing to the remarkable diversity of skeletal tissues [16, 17].

### 2.1 Intra‐ Versus Extrafibrillar Mineralization

The self‐assembly of collagens into homo‐ or hetero‐fibrils yields supramolecular structures that are generally regarded as a template for CaP biomineralization, serving as the scaffold where mineral formation is initiated. Collagen fibrils are also involved in diverse roles associated with regulatory processes in biomineralization, owing to their specific interactions with non‐collagenous proteins (NCPs) [18] and calcium ions [19]. These processes appear to underlie what is commonly referred to as “intrafibrillar mineralization,” which allows the loading of about 20%–40% of bone mineral [20, 21]. Intrafibrillar mineralization refers to the formation of CaP mineral within fibrils, which are composed predominantly of type I collagen. The latter consists of highly anisotropic triple‐helical polypeptide chains that self‐assemble into fibrils through a cell‐regulated process known as fibrillogenesis, yielding fibrils with a characteristic D‐periodicity of approximately 67 nm [22]. The mineralization process is often described as proceeding through the initial infiltration of ionic and/or amorphous mineral precursors into collagen fibrils, followed by the formation of amorphous calcium phosphate (ACP). ACP subsequently transforms into HAP, with crystallization occurring either within or outside the fibrils depending on the local physicochemical environment, particularly the presence of NCPs. Microscopic studies have, indeed, confirmed the presence of CaP in both intrafibrillar and interfibrillar regions [23]. However, the dynamics of this process have been investigated primarily using acellular in vitro models. Consequently, their relevance to native mineralized tissues remains difficult to establish because mineralization at the fibrils level is challenging to characterize while preserving both tissue architecture and chemical composition.

The mechanism of intrafibrillar mineralization provides a clear understanding of the nucleation and growth of CaP particles and thus supports the significant contribution of this mineralization to the mechanical properties of bone. It is also important to note the role of NCPs in this process. NCPs are mainly intrinsically disordered proteins (IDPs) whose role in mineralization remains a vast field of investigation [24] (for more details, see Section 2.3). However, it does not address what occurs next in the extrafibrillar space. Current in vitro models focusing on intrafibrillar mineralization fail to explore the potential for subsequent extrafibrillar mineralization, leaving the extensive mineralization in the extrafibrillar space unexplained. In mineralized tissues, the mineral fraction in the extrafibrillar space exceeds that within the collagen fibrils, with approximately 60%–80% of bone tissues [20, 21].

### 2.2 Matrix Vesicles

**FIGURE 1:**
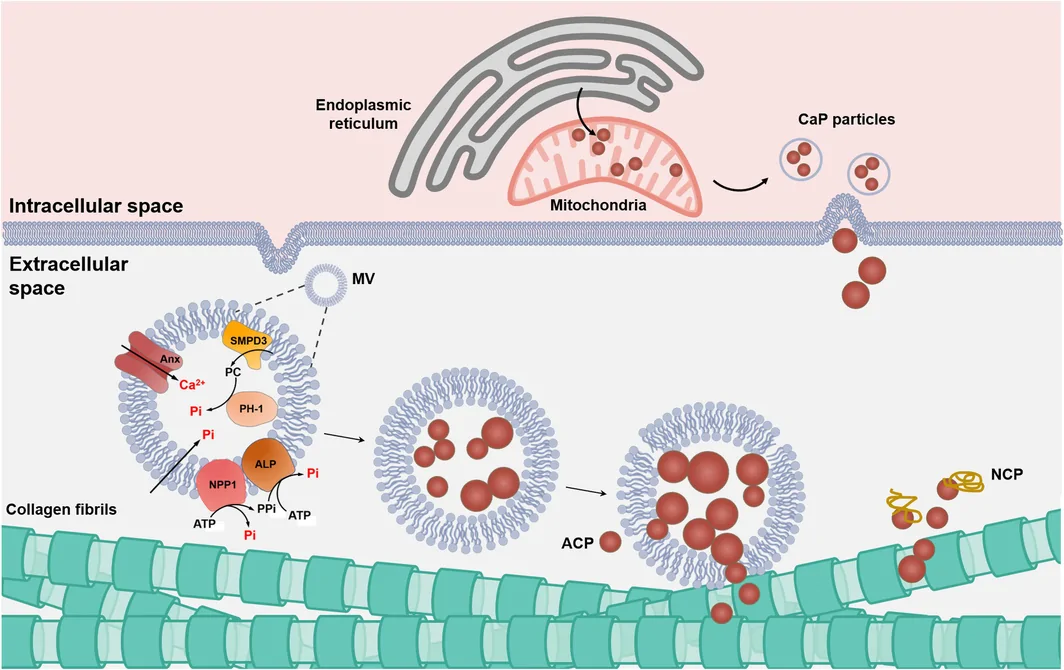
Proposed biomineralization pathways. Intracellular space: ER provides ions that precipitate within the mitochondria. CaP particles are then released in the ECM. Extracellular space: MVs are secreted by budding from the plasma membrane and accumulate calcium via Anx. Pi is generated at MV surface by NPP1 and TNAP enzymatic activities, while within MVs, sphingomyelin phosphodiesterase 3 (SMPD3) and phospho‐1 (PH‐1) cleave phospholipids releasing Pi. The accumulation of calcium and phosphate within the MV promotes precipitation and the formation of ACP. At the same time, mineralization occurs within and between collagen fibers in association with NCPs. Inspired from Refs [25, 26].

Although mineralization ultimately leads to the deposition of a mineral phase in the ECM, its initiation is thought to occur intracellularly through coordinated interactions between cellular organelles, including the endoplasmic reticulum (ER) and mitochondria (Figure 1) [25]. The ER has been proposed as the primary intracellular site for the accumulation of calcium and phosphate, which are then transferred to the mitochondria, where CaP particles approximately 30 nm diameters have been observed [27]. Recent work suggests that they are subsequently transferred into specialized extracellular vesicles (EV) before being released into the ECM [28].

Mineralizing cells such as hypertrophic chondrocytes, osteoblasts, odontoblasts, and “osteoblast‐like” vascular smooth muscle cells release matrix vesicles (MVs), a specialized subtype of EVs that initiates mineral formation [25, 26]. MVs provide a confined microenvironment that promotes CaP nucleation and growth, thanks to their specific content of mineralization‐related proteins, including tissue nonspecific alkaline phosphatase (TNAP), ectonucleotide pyrophosphatase/phosphodiesterase 1 (NPP1), PHOSPHO1, and annexins, which together regulate phosphate production, calcium accumulation, and intravesicular mineral formation (Figure 1) [26, 29].

The precise characterization of the mineral phase formed within MVs remains challenging because of their fragility following isolation and the potential transformation of the mineral phase during sample preparation. Transmission electron microscopy (TEM) has provided valuable insights into intravesicular mineral formation despite these technical limitations. TEM analyses have revealed calcium phosphate nodules within MVs released by murine osteoblasts, followed by the formation of mineral platelets in the ECM [30]. Depending on the biological model and imaging approach, the intravesicular mineral has been described as either needle‐like or spheroidal and poorly crystalline, suggesting that its morphology may vary with tissue type or maturation stage [31, 32]. Although important questions remain to be addressed, these findings support that MVs act as primary sites of CaP nucleation and early mineral growth.

### 2.3 Factors Influencing CaP Biomineralization

To gain deeper insights into CaP biomineralization, it is crucial to explore the factors that govern nucleation, growth, and possibly crystallization pathways. Intrinsic factors primarily relate to the concentrations of calcium and orthophosphate ions, which are the key precursors of CaP compounds. The concentrations of these ions are tightly regulated within a narrow range by homeostasis. Tables 1 and 2 provide approximate concentrations of calcium and phosphate ions in the adult human body for different compartments. Notably, the vast majority of calcium (> 99%) is stored in the skeleton, while only small amounts circulate in the bloodstream (2.2–2.7 mM), including calcium bound to plasma proteins and free calcium ions (1.1–1.3 mM) [29, 35]. Through complex regulatory mechanisms involving bones, intestines, and kidneys [29], these levels are consistently maintained within a narrow range, rarely fluctuating by more than 5% [36]. Intracellular calcium concentrations are much lower compared to extracellular levels, with approximately 100 μM in the ER and just 0.1 μM in the cytosol. This steep gradient, spanning nearly 10 orders of magnitude, is sustained by the coordinated action of calcium channels, pumps in the cell membrane, mitochondrial uptake, and calcium sequestration within the ER [37].

**TABLE 1:** Approximate content of calcium and phosphorus in human adult [33].

	Total content (g)	Skeleton (%)	Soft tissues (%)	Extracellular fluid (%)
Ca	1000	> 99	< 1	< 1
P	700	85	14–15	< 1

**TABLE 2:** Approximate content and distribution of calcium and inorganic phosphate ions [29, 30, 34].

Electrolyte	Plasma (mM)	Extracellular medium (mM)	Hypertrophic chondrocytes (mM)	Matrix vesicles (mM)
Ca^2+^	2.2–2.7	3.12–3.16	1.2–1.6	57–73
Pi	0.8–1.5	2.04–2.42	21–23	34–42

Phosphate metabolism is essential for cellular function, energy transfer, genetic stability, and skeletal integrity. In the human body, approximately 80% of phosphorus is stored in bones and teeth as CaP mineral, providing structural hardness and acting as the primary phosphate reservoir, while the remaining 20% is distributed in soft tissues and extracellular fluid [29]. Outside the skeleton, over 99% of the body's phosphorus is located within cells, with only a small portion present in the extracellular fluid. Of this extracellular phosphorus, about 28% exists as phosphate ions and comprises nonprotein‐bound (~20%) and free species (~80%), including various protonated molecules and complexes with calcium, magnesium, and sodium ions. According to many studies, the normal plasma Pi concentration is ranging from 0.8 to 1.5 mM [38].

In addition to their amounts, the activities of calcium and phosphate ions are influenced by physicochemical conditions including pH, ionic strength, and temperature, which in turn influence the mineral solubility [7]. In the human body, these conditions are, however, tightly regulated, ensuring that these variables remain within a physiologically balanced range.

Specific molecules may also interfere in the mineralization by either promoting or inhibiting the nucleation and/or the growth of CaP compounds. In this context, two prominent examples are highlighted herein: an inorganic molecule, PPi, and a distinctive family of proteins known as NCPs. PPi is a by‐product of numerous metabolic reactions that serves as a potent inhibitor for CaP mineralization through multiple mechanisms described in the literature [39]. It is, indeed, well established that the process of CaP mineralization is regulated by the molar concentration ratio of PPi to Pi, which is precisely modulated by the coordinated enzymatic actions of NPP1 and TNAP [26, 40] (Figure 1). On the other hand, a significant group of NCPs found in mineralized tissues is the Small Integrin‐Binding Ligand, N‐linked Glycoprotein (SIBLINGs) family, which plays a crucial role in regulating mineralization processes [41]. This protein family consists of five key proteins: osteopontin (OPN), bone sialoprotein (BSP), matrix extracellular phosphoglycoprotein (MEPE), dentin matrix protein 1 (DMP1), and dentin sialophosphoprotein (DSPP). These proteins are ubiquitous in mineralizing matrices and share several structural and functional characteristics [42]. Firstly, they are classified as IDPs, meaning they possess localized or global unstructured regions that provide exceptional flexibility, enabling them to fold and unfold dynamically upon binding to other molecules or minerals. Secondly, SIBLING proteins feature key functional domains, including a collagen‐binding domain, a HAP‐binding domain, and an Arg‐Gly‐Asp integrin‐binding motif, which collectively support their critical involvement in mineralization processes. Thirdly, these proteins undergo posttranslational phosphorylation, which further influences their activity. Depending on the context, SIBLING proteins can either promote or inhibit CaP mineralization, likely through a cooperative mechanism involving other actors such as enzymes, collagen, and other molecules [7, 42]. Despite their evident importance, the precise mechanisms through which SIBLING proteins regulate the nucleation and growth of the mineral phase remain poorly understood and appear to be highly dependent on the mineralized tissue among other factors [43]. Matrix gla protein (MGP) is another protein, which is also involved in CaP mineralization. It is a vitamin K‐dependent protein known to inhibit the mineralization, probably by binding calcium ions, as shown in MGP‐deficient mice [44].

Accordingly, investigating the mechanism of CaP biomineralization requires dealing with the complexity of the environment in which the mineral nucleates, grows, and eventually crystallizes within the tissue of interest. These processes are highly dependent on both physicochemical and physiological factors and involve various biochemical activities, such as enzyme‐catalyzed reactions, molecular interactions, ion transport activities, etc. [45]. As evidenced by the numerous studies detailed above, proteins remain key central and indispensable players in the process of biomineralization, which accounts for the significant attention they have received in the literature.

## 3 Interaction Between Nucleic Acids and CaP

Nucleic acids could also play a critical role in regulating the mineralization process, due to the remarkable diversity of their molecular structure, their capacity to interact with a wide range of biological entities, and their potential involvement in mechanisms directly associated with mineralization.

### 3.1 Nucleic Acids—CaP Coprecipitation

Since 1973, the precipitation of DNA using Ca^2+^ ions has been employed in nucleic acid transfection assays [46]. This method enhances transfection efficiency and offers greater resistance to DNase treatment compared to DEAE‐dextran‐based approaches. This has motivated the development of advanced transfection methods using preformed uni‐ or multi‐shell CaP nanoparticles as carriers for nucleic acids through a coprecipitation procedure. While these nanoparticles have shown lower transfection efficiency in 2D cell cultures compared to lipofectamine [47], they have demonstrated superior DNA delivery in 3D culture systems, with improved cellular penetration [48]. Furthermore, optimizing the Ca/P stoichiometry and mixing mode can enhance transfection efficiency, with a twofold increase when using controlled mixing [49].

Beyond DNA delivery, numerous studies have explored the use of nanoparticles to deliver small interfering RNA (siRNA) and microRNA (miRNA) in both in vitro and in vivo applications (see Ref. [50] and references therein). The CaP core of these nanoparticles dissolves in endosomes, resulting in the release of siRNA into the cytoplasm of target cells. This mechanism has proven highly effective for siRNA and miRNA transfection, as the incorporation of these molecules onto the nanoparticles enhances stability, reduces toxicity, and promotes long‐term expression of the delivered cargo [51].

From a mechanistic perspective, the coprecipitation of nucleic acids and CaP has received very little attention. The few available studies describe experiments in which DNA was mixed with calcium and phosphate ions in a buffer solution, with the aim of generating a DNA‐CaP precipitate and determining whether the presence of DNA influenced the nature of the mineral phase that formed. X‐ray diffraction (XRD) analysis did not reveal noticeable changes in the crystal structure of HAP grown with DNA. However, DNA appeared to slow HAP formation and induce slight changes in its lattice parameters, suggesting an interaction between DNA and the growing mineral phase during mineralization [52]. Nevertheless, the nature of this interaction remains unknown. More recently, CaP mineralization experiments have been conducted on DNA origamis, which exploit the programmable self‐assembly of DNA to create nanostructures with well‐defined geometries. These studies revealed that the mineral deposition is confined to the DNA architectures and reproduce their overall shapes, highlighting the ability of DNA to template CaP mineralization [53, 54]. In another study, quartz crystal microbalance measurements demonstrated that DNA‐coated surfaces accelerate heterogeneous CaP mineralization and promote the formation of a HAP‐like phase [55].

These observations indicate that DNA can act as a structural template for mineralization. In this mechanism, the geometry of the nucleic acid assembly constrains the spatial organization of the mineral phase. Consequently, CaP deposition reproduces the geometry imposed by DNA architectures rather than following the preferred crystallographic growth directions [53, 56, 57, 58, 59]. The precise molecular mechanisms underlying this templating effect, however, remain incompletely understood. All these studies suggest that nucleic acids can modify the CaP mineralization pathway and favor their incorporation into the mineral phase.

### 3.2 Nucleic Acids–CaP Interaction

DNA has been detected in ancient mineralized tissues, highlighting the remarkable ability of bone to preserve nucleic acids over extended periods [60]. This preservation relies on a combination of physicochemical, structural, and environmental factors. Bone can be regarded as a semi‐closed mineral matrix that slows the hydrolytic degradation of DNA, although the mechanisms underlying its preservation remain incompletely understood and can vary considerably even among bones recovered from the same environment [61]. Bone density and low porosity have consistently been associated with improved DNA preservation, possibly because they provide protected microenvironments that limit molecular degradation [62]. More recently, Goldberga et al. [63] used dynamic nuclear polarization (DNP)‐enhanced nuclear magnetic resonance (NMR) to demonstrate the presence of extracellular DNA (eDNA) in native mouse calvaria. This observation further supports the hypothesis that the long‐term preservation of DNA may result, at least in part, from its association with the mineral phase. Consistent with this hypothesis, several studies have highlighted specific interactions between nucleic acids and calcium phosphate (CaP) minerals [64, 65].

**FIGURE 2:**
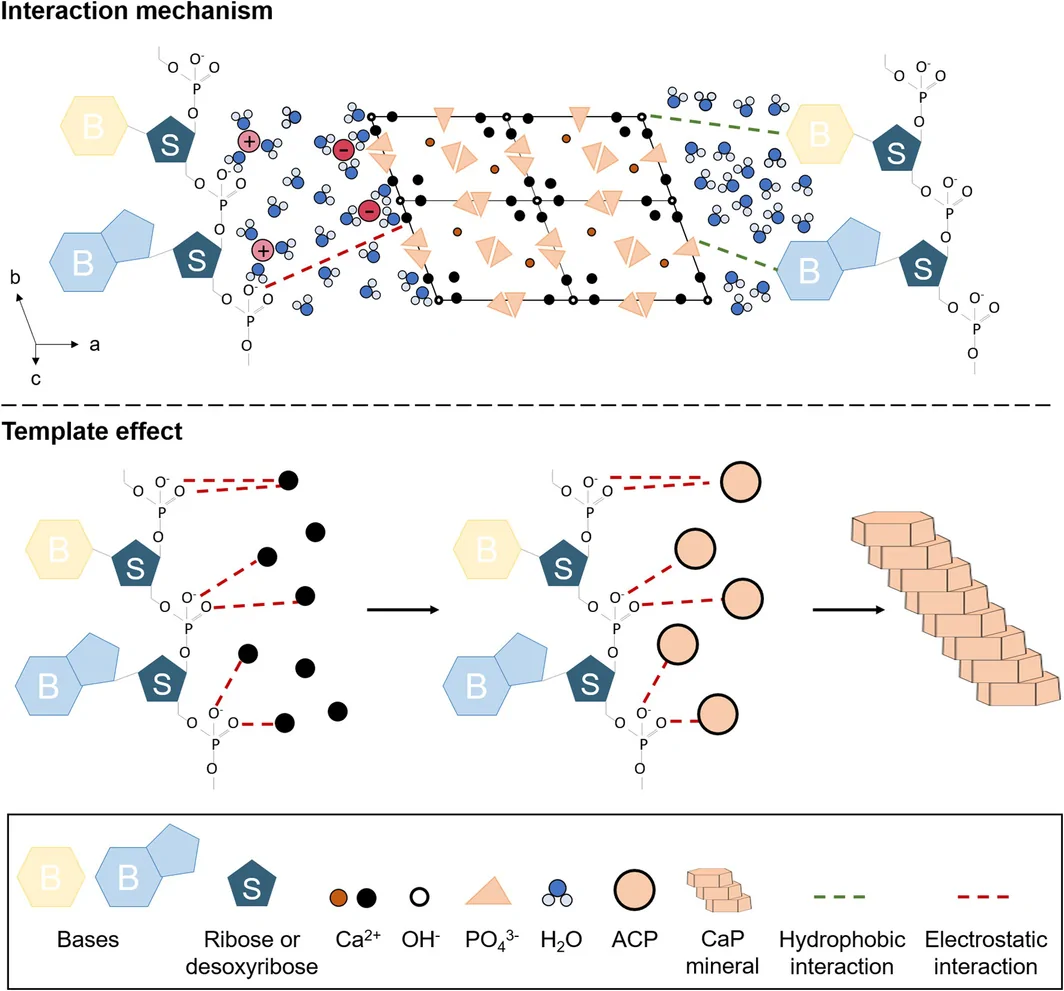
Proposed mechanism of the interaction between nucleic acid and CaP mineral and of the template effect of nucleic acids on CaP mineral growth. The interaction mechanism proposed that the phosphate groups of nucleic acids link HAP lattice through electrostatic and hydrophobic interactions. Nucleic acids can also serve as a template for CaP mineral growth through electrostatic interactions with calcium ions.

According to in silico studies, this interaction has been identified as mainly electrostatic, involving the calcium ions present on the HAP surface and the phosphate groups of DNA [66]. These findings are consistent with experimental studies showing that increasing the pH from 6.8 to 8.0, which causes deprotonation of phosphate groups, decreases the binding affinity of DNA to HAP [67]. Moreover, high salt concentrations diminished the binding of double‐stranded DNA to HAP by reducing electrostatic interactions. Conversely, single‐stranded DNA (ssDNA) binding was enhanced in the presence of salts, likely due to increased hydrophobic interactions [67]. These findings suggest that ssDNA could bind to HAP in a multilayer manner, where electrostatic bonds dominate the first layer, and hydrophobic interactions contribute to subsequent layers (Figure 2).

Other factors influence DNA conformation and its capacity to interact with CaP. Temperature has been shown to regulate this interaction, with lower temperatures (from 20°C to 25°C) enhancing ssDNA stability and its binding to HAP [67], and higher temperatures promoting the adsorption of longer RNA to HAP [68]. Interestingly, the nature of the mineral itself also impacts the interaction with nucleic acids. The stability of the B helix of DNA, evaluated by the temporal evolution of the root mean square deviation between DNA atoms, was higher when adsorbed to HAP. However, when DNA was adsorbed to calcium oxalate (CaOx), the helix was completely destabilized. This effect was attributed to the repulsion effect between Ox^2−^ and the DNA phosphate backbone, leading to the separation of the two strands [69].

The modifications in DNA sequence could also affect the binding to CaP. The systematic evolution of ligands by exponential enrichment (SELEX) method was employed to identify the most efficient DNA aptamers for CaP synthesis. Intriguingly, it was revealed that they comprised a high percentage of guanine nucleotides, with the variable region of aptamers of 40 bp exhibiting approximately 43% ± 9% of guanine content [70]. Additionally, these aptamers exhibited a G‐quadruplex structure that was further stabilized in the presence of sodium chloride [71].

Important question is whether the effects described above are specific to nucleic acids or simply reflect the behavior of polyanionic macromolecules. Indeed, electrostatic interactions between phosphate groups and calcium ions are not unique to DNA or RNA and are also observed in other polyanions of biological relevancy, including polyphosphates [72], glycosaminoglycans [73], and acidic polypeptides [74]. Therefore, part of the ability of nucleic acids to interact with CaP may simply arise from their high density of negatively charged phosphate backbones (Figure 2). However, electrostatic interactions alone cannot fully explain nucleic acid–CaP interactions. Salt‐dependent modulation of DNA binding to HAP indicates that hydrophobic interactions involving exposed nucleobases also contribute to nucleic acid–CaP interactions [67]. Moreover, SELEX experiments identified CaP‐binding aptamers enriched in guanine residues, suggesting that nucleotide composition influences mineral affinity [70]. Likewise, the stabilization of G‐quadruplex structures and the ability of DNA origami architectures to direct crystal growth indicate that higher‐order nucleic acid structures can affect mineralization [71]. Together, these findings indicate that nucleic acid–CaP interactions arise from the combined contribution of multiple weak interactions, including electrostatic and hydrophobic interactions, with hydrogen bonding likely providing an additional contribution (Figure 2). Their relative importance is further modulated by environmental conditions, nucleotide sequence, and higher‐order nucleic acid structures. Although the relationship between RNA and biomineralization remains poorly explored, RNA is expected to interact with CaP through similar physicochemical mechanisms because of its structural similarity to DNA and the presence of a negatively charged phosphate backbone.

## 4 Potential Involvement of Nucleic Acids in Biomineralization

### 4.1 Extracellular Nucleic Acids

**FIGURE 3:**
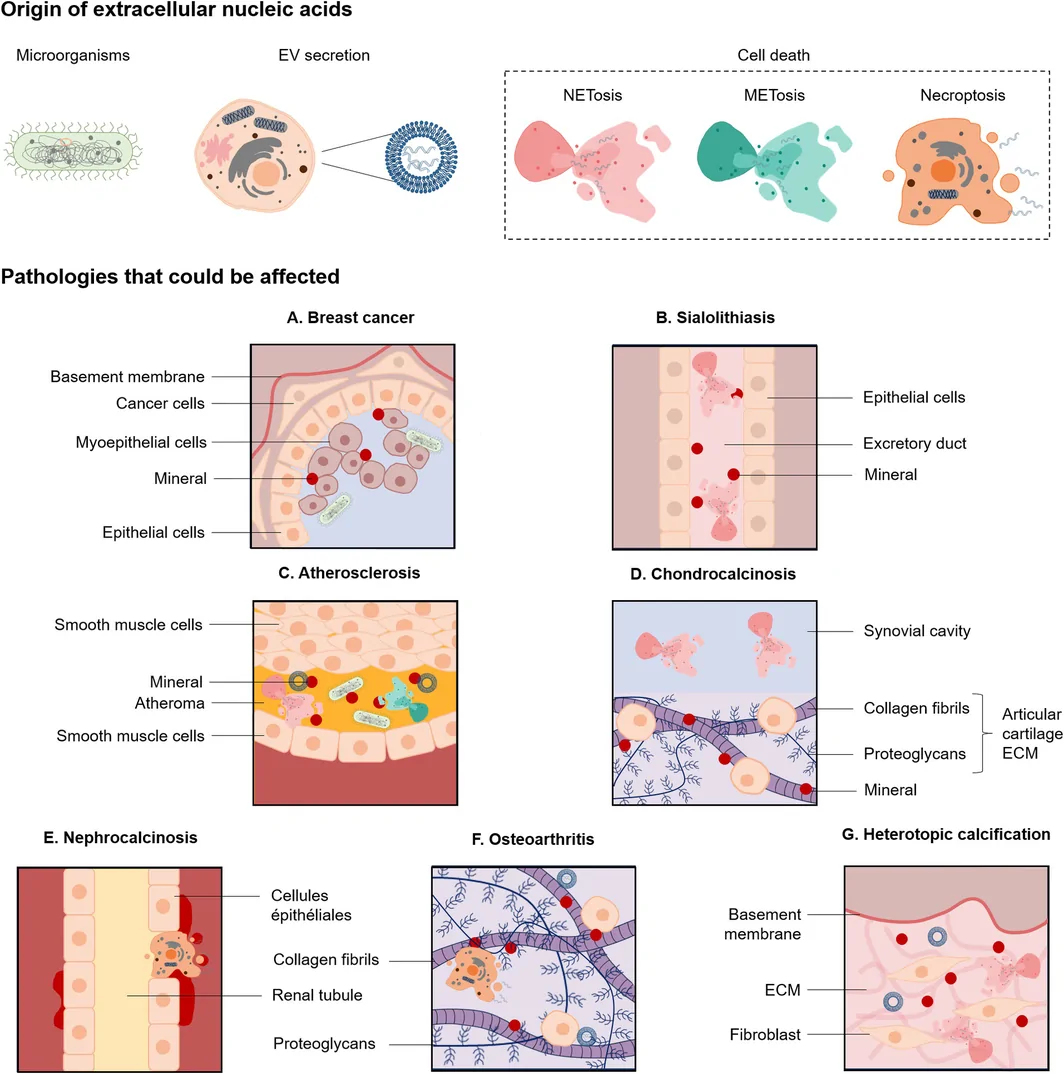
Extracellular nucleic acids: origins and potential involvement in pathological calcification. Extracellular nucleic acids originate from multiple biological sources, including microorganisms, extracellular vesicle (EV) secretion, and cell death processes such as neutrophil extracellular trap formation (NETosis), macrophage extracellular trap formation (METosis), and necroptosis. Extracellular DNA and RNA may accumulate within the extracellular matrix (ECM) and interact with calcium phosphate minerals, potentially contributing to pathological mineralization. Representative examples of diseases in which extracellular nucleic acids and ectopic calcification may coexist include (A) breast cancer, (B) sialolithiasis, (C) atherosclerosis, (D) chondrocalcinosis, (E) nephrocalcinosis, (F) osteoarthritis, and (G) heterotopic calcification.

At a first glance, a possible involvement of nucleic acids in biomineralization processes presupposes their presence within mineralized tissues as extracellular nucleic acids. Demonstrating such a presence is challenging, particularly because extracellular nucleic acids are difficult to distinguish from intracellular counterparts. As described before, nucleic acids have been identified in native mouse calvaria [63]. Isotopic labeling in heavy mice enabled them to be precisely localized in the ECM and distinguished from cellular nucleic acids. The signals observed further indicated that these nucleic acids did not originate from cell lysis, as their well‐structured organization suggested anchorage in the ECM [63]. Nucleic acids have also been detected in a variety of mineralized tissues, in pathologies such as atherosclerosis [75], sialolithiasis [76], or breast cancer [77]. Extracellular nucleic acids can originate from several processes (Figure 3).

- Cell‐free nucleic acids (cfNAs), first identified by Mandel and Metais in 1948 in human blood plasma [78], are cell‐independent nucleic acids that circulate in body fluids. cfNAs are small molecules typically around 80–200 bp in length, comprising both DNA and RNA, and are naturally present in biological fluids at concentrations estimated to range from 10 to 30 ng/mL [79]. cfNAs have been closely linked to various diseases, such as lupus erythematosus [80], cancers [81], and chromosomal disorders [82], and are used in diagnosis as biomarkers.
- EVs, which serve as molecular cargo, contain nucleic acids. While their DNA content, including genomic and mitochondrial DNA, is rare, EVs more frequently carry various forms of RNA, especially small RNAs. The types of RNA found in EVs include messenger, micro, transfer, ribosomal, long noncoding, small noncoding, piwi‐interacting, vault, and Y RNAs [83].
- Neutrophils, the most common immune cells, are the human body's first line of defense. Their capacity to fight pathogens involves a suicide process called NETosis, characterized by the release of neutrophil extracellular traps (NETs), which facilitate the trapping and subsequent neutralization of invaders. This property has been discovered by Takei et al. in 1996 [84]. Similarly, macrophages produce macrophage extracellular traps (METs) under certain conditions [85]. Both NETs and METs are composed of nuclear DNA, mitochondrial DNA, histones, and additional proteins derived from nuclear content [85, 86].
- Another source of extracellular nucleic acids is necroptosis, a programmed cell death exhibiting the characteristics of both apoptosis and necrosis, which triggers an inflammatory response. Necroptosis is characterized by the loss of cell membrane integrity and the release of cellular content, including DNA [87].
- Microorganisms are also a possible source of extracellular nucleic acids. The release of nucleic acids from microorganisms, which include bacteria, eukaryotes, and viruses that compose the microbiota, is a well‐documented phenomenon [88].

### 4.2 Pathological Mineralization

The biological mechanisms behind the presence of extracellular nucleic acids are observed in physiological conditions, but are generally amplified in pathological conditions. Pathologies involving biomineralization defects occur not only in mineralized tissues, but also in soft tissues. Ectopic mineralization of soft tissues is observed in a variety of pathological conditions and can affect most tissues (Table 3). Unlike physiological biomineralization, pathological mineralization in mammals can lead to the formation of various crystals, primarily CaP, but also calcium pyrophosphates (CaPP), CaOx, calcium carbonates, monosodium urate (MSU), and other minerals often associated with organic compounds [96]. Cells play a crucial and direct role in the process even though their active implication remains unclear. Various cell types may be implicated, depending on the tissue affected, differing from those involved in physiological mineralization [97]. The variety of mineral types, their sizes, localizations, and distributions formed during pathological mineralization makes it impossible to generalize about the formation mechanism. A comprehensive study of mineral characteristics, including inorganic and organic composition, structure, and morphology, is thus essential to better understand each pathophysiology [97].

**TABLE 3:** Examples of tissues affected by ectopic mineralization.

Tissue		Mineral types	Disease	References
Blood vessel	Intima	CaP	Atherosclerosis	[89]
	Media	CaP	Atherosclerosis Chronic kidney disease	[90]
Myocardium		CaP	Mitral annular calcification	[89]
Heart leaflets		CaP	Calcific aortic stenosis	[91]
Articular cartilage		CaP, CaPP	Osteoarthritis, chondrocalcinosis	[92, 93]
Eye		CaP	Sclerochoroidal calcification, corneal and conjunctival calcifications	[122]
Kidney		CaPP, CaOx	Nephrocalcinosis	[94]
Salivary glands		CaP	Sialolithiasis	[95]
Breast tissue		CaP, CaOx	Breast cancer	[123]
Ovary		Psammoma bodies	Serous papillary adenocarcinomas	[124]
Thyroid			Papillary carcinomas	
Meninges			Meningioma	
Testis			Ovarian‐like epithelial tumor of the testis	[125]
Lungs			Primary acinic cell carcinoma, lung adenocarcinoma	[126]
Endometrium			Endometrial adenocarcinoma	[127]
Tendons		CaP, MSU	HAP deposition disease, Gout	[128]
Ligaments		CaP	HAP deposition disease	
Gallbladder		Calcium carbonate, calcium hydrogen bilirubinate	Gallstones	[129]

Among the pathologies in which abnormal mineralization occurs, some are directly associated with genetic mutations (Table 4). Most of the mutated genes encode proteins known to be implicated in the regulation of mineralization, in phosphate homeostasis or in bone and skeleton development. Interestingly, in Aicardi–Goutières syndrome, inactivating mutations in TREX1, which encodes the major cytosolic 3′–5′ exonuclease, are associated with calcification in the basal ganglia [95]. In this disease, the accumulation of cytosolic nucleic acids may contribute to the ectopic mineralization process observed.

**TABLE 4:** Mutations inducing diseases exhibiting an ectopic calcium‐related mineralization. Cancers exhibiting ectopic mineralization linked to mutations have not been listed.

Disease	Gene mutated	Altered functions	Phenotypic mineralization features	References
Spondyloepiphyseal dysplasia with precocious OA	Col2A1	Skeleton development and cartilage resistance	Early onset OA	[130]
Fibrodysplasia ossificans progressive (FOP)	ACVR1	Bone morphogenetic protein signaling	Ossification in skeletal muscle, tendons, ligaments, fascia, and aponeuroses	[131]
X‐linked chondrodysplasia punctata	ARSE	Cartilage and bone matrix development	Larynx, trachea, and brunchi calcifications	[132]
Chondrocalcinosis/calcium pyrophosphate deposition disease/pseudogout	ANKH	ATP transport	Mineral deposition in joints	[133]
Generalized arterial calcification in infancy/idiopathic infantile arterial calcification (GACI)	ENPP1 ABCC6	PPi generation ATP transport	Vascular calcification, joint and spine ossification	[134]
Pseudoxanthoma elasticum (PXE)	ABCC6	ATP transport	Vascular, dermal, and eye mineralization	[134]
Idiopathic infantile hypercalcemia type 2	SLC34A1	Phosphate transport	Kidney stones	[135]
Fahr's syndrome/idiopathic basal ganglion calcification	SLC20A2, XPR1, PDGFRB, or PDGFB	Phosphate homeostasis	Vascular and pericapillary calcifications in the brain	[134]
Werner syndrome	WRN helicase	Magnesium and ATP‐dependant helicase	Tendons, ligaments, and subcutaneous calcifications	[136]
Keutel syndrome	MGP	Mineralization inhibition	Vascular and cartilage calcification	[137]
X‐linked hypophosphatemia	PHEX	SIBLING regulation	Vascular, tendon, and ligament calcifications	[138]
Raine syndrome	FAM20C	Biomineralization regulation	Intracranial calcifications	[139]
Enamel renal syndrome	FAM20A	Teeth biomineralization regulation	Intrapulpal calcifications	[140]
Arterial calcification due to deficiency of CD73	NT5E	Nucleotide monophosphase	Vascular and joint calcifications	[141]
Aicardi‐Goutières syndrome	RNASEH2B, TREX1, SAMHD1, RNASEH2C	Ribonuclease, exonuclease, deoxynucleoside triphosphate	Basal ganglia calcifications	[139]
Augustine‐null blood type	SLC29A1	Nucleoside transport	Joint calcifications	[142]
KARS‐related progressive leukoencephalopathy	KARS	Attachment of amino acid to tRNA	Brain and spinal cord calcifications	[143]
Kenny‐Caffey syndrome 2	FAM111A	DNA‐binding serine protease	KCS1‐like phenotype and corneal and retinal calcifications	[144]
Cockayne syndrome	ERCC6 ERCC8	Nucleotide excision	Intracranial calcifications	[139]
β‐thalassemia/sickle cell anemia	HBB	Oxygen transport	Vascular, dermal, and eye mineralization	[145]
Hutchinson‐Gilford progeria syndrome	LMNA	Nuclear organization	Vascular calcifications	[134]
NRROS‐associated neurodegeneration	NRROS	TGFB1 regulation	Calcifications in subcortical and periventricular white matter	[143]
Primrose syndrome	ZBTB20	Hematopoiesis, oncogenesis and immune response	Calcification of the external ear and brain parenchyma	[146]
Mitochondrial myopathy, encephalopathy, lactic acidosis, and stroke‐like episodes	MTTL1	Mitochondrial metabolism	Bilateral basal ganglia calcifications	[143]
Progressive osseous heteroplasia	GNAS locus	Parathyroid hormone regulation	FOP‐like changes and dermal ossifications	[131]
Albright hereditary osteodystrophy			Subcutaneous ossifications	[147]
Osteoma cutis				
Pseudohypoparathyroidism				
Pseudopseudohypoparathyroidism				
Papillon‐Lefevre syndrome	CTCS	Immune system proteases	Intracranial calcifications	[139]
Singleton‐Merten syndrome 1	IFIH1 or DDX58	Innate immunity	Vascular calcifications and skeletal abnormalities	[134]
Gitelman syndrome	NCCT TRPM6	Electrolyte homeostasis	Sclerochoroidal calcifications	[148]
Gorlin‐Goltz syndrome/basal cell nevus syndrome	PTCH1	Sonic, Indian, and Desert hedgehog signaling	Intracranial calcifications	[139]
PXE‐like syndrome/multiple vitamin K‐dependent coagulation factor deficiency	GGCX	Gla residues carboxylation	Dermal mineralization	[145]
Gaucher disease	GBA	Glycosylceramide catabolism	Vascular calcifications	[134]
Greenberg dysplasia	LBR	Cholesterol synthesis	Polydactyly and chondro‐osseous changes	[148]
SHORT syndrome	PIK3R1	Endocytic trafficking	Kidney stones	[149]
Kenny‐Caffey syndrome 1 (KCS1)	TBCE	Tubulin folding pathway	Intracranial calcifications	[147]
Primary hyperoxaluria type 1	AGT	Glycoxylate detoxification	Calcifications in renal tubular cells, interstitium, or tubular lumen, and kidney stones	[151]
Medullary sponge kidney	GDNF RET	Neuronal cell migration, proliferation, and differenciation	Calcifications in renal tubular cells, interstitium, or tubular lumen, and kidney stones	[152]

Pathological mineralization could also involve extracellular nucleic acids, as the presence of extracellular nucleic acids has been reported in calcifications. Microcalcifications are indeed observed in breast cancer (Figure 3A); their detection by mammography enables early diagnosis of the disease [98]. Two types of calcifications have been identified: type I, formed of CaOx and type II, composed of HAP. Analysis of type II calcifications using Raman spectroscopy showed the presence of eDNA encapsulated and adsorbed to HAP in most samples [77]. This suggests the potential modulatory role of DNA in an ectopic biomineralization beyond an adsorptive process [77]. eDNA is also observed in sialoliths [76], which are salivary gland stones secreted mainly by the submandibular gland, but by the parotid gland (Figure 3B) [99]. Sialoliths are composed of calcium associated with proteins and other minerals generally formed by HAP or whitlockite [100]. The clinical manifestations of sialolithiasis are a postprandial pain and recurrent bacterial infections. The etiology of sialolithiasis remains poorly understood. While initial dysfunction of the secretory glands is considered the most plausible reason, it has also been suggested that foreign bodies, possibly bacteria, could provide a nest for crystal growth [101]. Schapher et al. [76] identified that most sialoliths contain bacterial DNA coming from the common oral and intestinal microbiome. Their findings have been confirmed by other studies, which also suggest the involvement of bacteria in the development of sialolithiasis [102]. Similarly, microcalcifications in human atheromatous lesions of the aorta and femoral artery contain eDNA [75], which may in part be of bacterial origin. Indeed, bacteria, particularly of periodontal origin, are commonly found in vascular lesions [89]. In addition to bacteria, nanobacteria, which are replicating entities of controversial nature, have been identified in the psammoma bodies of serous adenocarcinomas of the ovary, in kidney stones and in calcified human arteries and cardiac valves, suggesting either a passive adsorption process or their potential contribution to calcification formation [103, 104].

Calcifications associated with eDNA could also result from activation of the immune system in response to infection or to tissue damage. In addition to the presence of bacterial DNA in sialoliths, Schapher et al. [76] showed that NETs play an essential role in the growth of sialoliths, suggesting that the microbiome could stimulate the production of NETs. Infective endocarditis is an infection of the heart endothelium, typically caused by *Staphylococcus aureus* or *Viridans streptococci*, and often associated with calcification of mitral annulus [105]. It is characterized by bacterial adhesion to the endothelium, possibly followed by internalization. This triggers a major immune response involving the formation of NETs, leading to thrombus formation [106]. Neutrophils and NETs have also been reported in atheromatous lesions [107], as macrophages, which are notably involved in foam cell formation [108]. In addition to NETs, METs, containing DNA, have also been observed in atherosclerosis (Figure 3C) [85]. Their role in vascular calcification remains to be determined, but they seem also to be involved in arterial thrombus formation [109] and in the maintenance of inflammation via DNA activation of NLRP3 inflammasome [92]. The association between eDNA, inflammation and calcification is well illustrated in the process of heterotopic ossification, which refers to the formation of extra‐skeletal bone in soft tissue (muscles, tendons, ligaments, or fascia), usually following traumatic injury, burns, or surgery [110]. Heterotopic ossification is associated with an intense immune response characterized by neutrophil activation and NETosis. Interestingly, by treating mice with an antagonist of TLR9, an eDNA receptor involved in NETosis, heterotopic ossification was significantly reduced [111]. This study highlights the role of NETosis in heterotopic ossification, which may involve the biomineralization capacity of eDNA. Chondrocalcinosis also associates calcifications, inflammation, and NET formation (Figure 3D). Chondrocalcinosis, also known as pseudogout or CaPP deposition disease, is characterized by a painful swelling of articular joints. It differs from the gout in the type of crystal deposition [94]. In chondrocalcinosis, the crystal formation induces a significant inflammation characterized by the recruitment of immune cells such as neutrophils [93]. Pang et al. [112] demonstrated that CaPP induce the formation of NETs in vitro, suggesting that NET formation could be secondary to that of CaPP and may contribute to the persistence of inflammation and, potentially, to cyst growth through the release of eDNA.

Another cell death releasing eDNA is necroptosis. Necroptosis is observed in nephrocalcinosis and osteoarthritis (OA), two diseases associated with ectopic mineral deposition (Figure 3E,F). In nephrocalcinosis, CaP and CaOx mineralization is most often localized in the renal medulla. CaP and CaOx crystals induce necroptosis [113], releasing eDNA that may be able to regulate other minerals' formation leading to a runaway reaction. In OA, CaP is observed in articular cartilage and its extent is associated with OA progression [114]. Chondrocyte death, which is strongly associated with cartilage degradation [115], is observed in the area of cartilage mineralization [116]. Specifically, chondrocytes can undergo necroptosis [115, 117], that could be a new player in articular cartilage mineralization in OA.

Apoptosis is also involved in the mineralization process, trough the generation of apoptotic bodies. Smooth muscle cells (SMC) apoptosis is observed with numerous apoptotic body release in the intima of atherothrombotic arteries [1, 99]. Coscas et al. [75] colocalized eDNA of microcalcification areas with dead cells in human early‐stage atheromatous aorta. SMCs also follow a transdifferentiation process toward an osteoblast‐like phenotype, characterized by the downregulation of the alpha‐smooth muscle actin and the upregulation of osteoblast markers, such as the transcription factor RUNX2 [118]. Osteoblasts mineralize their matrix thanks to the release of MVs. MVs contain nucleic acids, which may play a role in their ability to cause intravesicular calcifications [119]. As observed in the atherothrombotic vessel wall for SMCs, chondrocytes undergo phenotype changes in OA cartilage, including hypertrophic differentiation. Hypertrophic chondrocytes are found physiologically in the calcified cartilage layer of the articular cartilage and in the growth plate cartilage, where they mineralize cartilage matrix, which is crucial for the replacement of cartilage by bone in the endochondral ossification process of long bone growth. In OA cartilage, the messenger RNA expression of type X collagen, a marker of hypertrophic chondrocytes, is correlated with the extent of mineralization [114]. Hypertrophic chondrocytes release MVs, involved in cartilage mineralization [26, 120]. MVs can also be involved in CaPP formation in chondrocalcinosis [94]. MVs are specific EVs that anchor themselves in the ECM and participate in the mineralization process. In many pathologies where ectopic mineralization occurs, EVs have been found. For example, EVs have been identified in heterotopic ossification [110] and have been shown to be associated with calcifications in vascular diseases [121] or in calcific tendonitis [34] (Figure 3G). Their nucleic acid content may play a role in these ectopic mineralizations.

Although eDNA has been observed in calcifications and is likely to be present in lesions associated with ectopic mineralization, and although accumulating evidence supports a role for NETs in disease progression [90], little evidence points to the actual involvement of eDNA in the mineralization process. However, a DNase treatment resulted in the abrogation of lesion formation in an ApoE−/− mouse model of atherosclerosis [91]. Furthermore, when injected into rats developing aortic aneurysm after the local perfusion of elastase, free DNA induces aortic calcification [75]. No calcification was observed in the absence of free DNA injection.

To provide a clearer overview of the field, we classified the pathological conditions discussed in this review according to the strength of the available data supporting a role for nucleic acids in ectopic mineralization (Table 5). While the current literature is largely based on correlative observations, mechanistic and causal data support the active involvement of nucleic acids in pathological mineralization and highlight promising directions for future research.

**TABLE 5:** Correlative, mechanistic, or causal link between extracellular nucleic acids and ectopic mineralization in pathological contexts.

Pathology	Observations	Link
Breast cancer	eDNA directly found in ectopic minerals	Correlative
Sialolithiasis	eDNA directly found in ectopic minerals NETs increase mineral growth	Mechanistic
Infective endocarditis	NETs found	Correlative
Atherosclerosis	Extracellular vesicles, NETs, and METs found eDNA directly found in ectopic minerals DNase treatment reduces ectopic mineralization	Causal
Heterotopic ossification	Extracellular vesicles and NETs found Inhibiting eDNA receptor reduces pathology	Mechanistic
Chondrocalcinosis	Extracellular vesicles and NETs found	Correlative
Nephrocalcinosis	Necroptosis happens	Correlative
Osteoarthritis	Extracellular vesicles found Necroptosis happens	Correlative

## 5 Conclusion and Outlooks

Biomineralization has traditionally been viewed as a process governed by the coordinated action of mineral ions, extracellular matrix proteins, cellular activities, and tightly regulated physicochemical conditions. The studies reviewed here confirm the central importance of these established regulators while revealing that this framework may still be incomplete. Although their role remains incompletely understood, increasing evidence suggests that nucleic acids represent an additional class of biomolecules capable of influencing CaP mineralization through mechanisms that extend beyond their canonical roles in genetic information storage and transfer.

Experimental studies have demonstrated that nucleic acids may interact with calcium phosphate minerals through a combination of electrostatic, hydrophobic, and likely hydrogen‐bonding mechanisms. Their ability to adsorb onto CaP surfaces, influence calcium phosphate precipitation, modify crystal growth and, in engineered systems, act as structural templates for mineralization indicates that they possess intrinsic physicochemical properties compatible with a regulatory role in biomineralization. Importantly, these interactions appear to depend not only on environmental conditions but also on nucleic acid sequence, conformation, and supramolecular organization, suggesting a level of specificity that distinguishes them from other negatively charged biological polymers.

These physicochemical observations gain particular significance in light of the data showing that extracellular nucleic acids are components of mineralized tissues. Extracellular nucleic acids originate from diverse cellular processes, including EV secretion, NETosis, necroptosis, and microbial activity. Their presence within the ECM therefore creates numerous opportunities for interactions with mineral precursors and nascent mineral phases throughout tissue mineralization.

Pathological mineralization provides particularly compelling evidence supporting this concept. Across a wide spectrum of diseases, extracellular nucleic acids are repeatedly associated with ectopic mineral deposits. Although many of these observations remain correlative, several experimental studies indicate that eDNA is not merely incorporated into calcifications but can actively modulate their formation. These findings suggest that extracellular nucleic acids may constitute a mechanistic interface linking inflammation, cell death, microbial colonization, and mineral deposition, thereby providing a conceptual framework capable of explaining common features shared by otherwise distinct calcification disorders.

Despite these advances, major challenges remain. One of the foremost questions is whether nucleic acids directly regulate physiological biomineralization or whether their functions become significant only under pathological conditions characterized by excessive extracellular nucleic acid release. Likewise, the respective roles of DNA and RNA, of nuclear, mitochondrial, and bacterial DNA, the contribution of EVs compared with free nucleic acids, and the influence of sequence, chemical modifications, and higher‐order structures on mineral formation remain largely unknown. Future studies should further determine how nucleic acids cooperate with established regulators of biomineralization, including collagen fibrils, MVs, SIBLING proteins, and PPi metabolism, and whether they contribute differently to intra‐ and extrafibrillar mineralization. Addressing these questions will require integrated multidisciplinary approaches capable of establishing causal rather than correlative relationships.

Beyond their mechanistic interest, extracellular nucleic acids may also open new translational perspectives. Their accessibility in biological fluids and calcified tissues makes them attractive candidates as biomarkers of mineralization activity, while therapeutic strategies targeting extracellular nucleic acids, NET formation, or EV biology could represent innovative approaches to limit pathological calcification while preserving physiological mineralization.

Overall, this review supports a conceptual framework in which nucleic acids emerge as candidate regulators of biomineralization acting at the interface between mineral chemistry, ECM biology and inflammatory signaling. Although the available evidence does not yet establish nucleic acids as bona fide components of the vertebrate biomineralization toolkit, integrating nucleic acids into current models of biomineralization opens a new research avenue with important implications for skeletal biology, pathological calcification, and biomaterials science. Future studies deciphering how nucleic acids cooperate with proteins, lipids, polysaccharides, EVs, and mineral precursors will likely contribute to a more comprehensive and unified model of calcium phosphate biomineralization in both physiological and pathological contexts.

## Author Contributions

Jessem Landoulsi and Xavier Houard conceived and designed the review. All authors were involved in drafting and revising the manuscript.

## Funding

This work was funded by The Emergence Program at Sorbonne University. Fanny Duhalde was supported by a PhD grant from the Ministère de l'Enseignement Supérieur et de la Recherche.

## Conflicts of Interest

The authors declare no conflicts of interest.

## Data Availability

Data sharing is not applicable to this article as no datasets were generated or analyzed during this study.
